# Factor XIII deficiency: an updated approach to diagnosis and management

**DOI:** 10.3389/fmed.2026.1895924

**Published:** 2026-08-18

**Authors:** Rahaf Mahmoud Altahan

**Affiliations:** 1 Hematology Unit, Pathology and Clinical Laboratory Medicine Administration, King Fahad Medical City, Riyadh Second Health Cluster, Riyadh, Saudi Arabia; 2 College of Medicine, Alfaisal University, Riyadh, Saudi Arabia

**Keywords:** congenital factor XIII deficiency, acquired factor XIII deficiency, factor XIII, congenital bleeding disorders, rare bleeding disorders

## Abstract

Factor XIII deficiency (FXIIID) is a rare bleeding disorder that may be congenital or acquired and is associated with significant morbidity, particularly in its congenital form, including intracranial hemorrhage, impaired wound healing, and adverse pregnancy outcomes. Unlike other coagulation factor deficiencies, FXIIID is not detected by routine coagulation screening tests, contributing to delayed diagnosis and underrecognition. This review provides an updated overview of the epidemiology, molecular biology, clinical manifestations, laboratory diagnosis, and management of FXIIID. Particular emphasis is placed on contemporary diagnostic strategies, including the advantages and limitations of functional, antigen, inhibitor, and genetic assays, as well as preanalytical and analytical pitfalls that may affect test interpretation. Special attention is given to acquired FXIIID, emerging diagnostic challenges, and complex clinical scenarios, including pregnancy, neonatal care, surgery, and anticoagulation. Persistent knowledge gaps, including assay standardization and limited evidence supporting current recommendations for managing special scenarios, are also highlighted. Continued international collaboration and prospective data collection are needed to improve diagnosis, refine treatment strategies, and strengthen future evidence-based guidance.

## Introduction

Coagulation factor XIII (FXIII), historically termed the fibrin-stabilizing factor (FSF), was first described by Robbins in the 1940s through studies of fibrin clot stability (1). Subsequent work by Laki and Lorand in the late 1940s further characterized this factor and demonstrated its role in rendering fibrin resistant to dissolution. During the 1950s, Lorand and others further elucidated its function in fibrin cross-linking and stabilization (2, 3). The first patient with FXIII deficiency (FXIIID) was described by Duckert and colleagues in Switzerland in 1960. Three years later, the FSF was formally designated *factor XIII* following the standardization of coagulation factor nomenclature (4, 5).

FXIII is a transglutaminase that plays a crucial role in blood coagulation by cross-linking fibrin molecules, thereby enhancing the mechanical strength of fibrin and its resistance to fibrinolysis. It circulates in plasma as a heterotetramer composed of two catalytic A subunits and two carrier B subunits (A_2_B_2_). Beyond coagulation and fibrin stabilization, FXIII is involved in extracellular matrix interactions, wound healing, angiogenesis, placental implantation, and clot remodeling (6, 7).

FXIIID can be congenital or acquired. Congenital deficiency can be quantitative (deficiency of A or B subunits) or qualitative (defective A subunits); in its severe form, it is associated with life-threatening hemorrhagic complications, mainly intracranial hemorrhage. Acquired forms are increasingly recognized across a broad range of disorders and can be either immune- or non-immune-mediated, with variable severity and treatment approach.

Despite the potentially severe bleeding complications, diagnosis is often delayed by false reassurance from normal coagulation screening tests, with detrimental consequences for patient outcomes. FXIII concentrates remain the standard-of-care replacement therapy in severe cases. However, if unavailable, fresh-frozen plasma and cryoprecipitate can be used as alternatives, albeit with inferior efficacy and safety (4).

This review provides an updated and comprehensive appraisal of the current understanding of FXIIID, encompassing its molecular basis, diagnostic approach, including the pitfalls that contribute to delayed recognition, and the full spectrum of management strategies across congenital and acquired disease, with particular attention to special clinical scenarios that remain incompletely addressed in the existing literature. It may serve as a practical resource for clinicians and laboratorians involved in the diagnosis and management of this rare but clinically significant disorder.

## Epidemiology

The estimated global prevalence of severe congenital FXIIID is approximately 1 in 1–3 million individuals (8, 9), with mild deficiency being considerably more common, estimated at 0.02%–0.79% (6, 10), though these figures likely underestimate the true burden of the disease.

As with other autosomal recessive disorders, prevalence is substantially higher, up to tenfold, in populations with high rates of consanguinity, particularly in the Middle East and South Asia (7), and has also been observed at increased rates in certain populations due to founder effects, including Switzerland (5, 11, 12). Underdiagnosis is a recognized and significant challenge, driven by two compounding factors: first, routine coagulation screening tests do not detect FXIIID (see Section “Diagnostic pitfalls”); and second, relatively low FXIII levels can maintain adequate hemostasis, meaning that individuals with mild deficiency may remain asymptomatic until exposed to hemostatic stress, and therefore go unidentified. Accordingly, FXIIID is considered one of the most underdiagnosed bleeding disorders (13).

Autoimmune acquired FXIIID is exceptionally rare, with approximately 100 cases reported worldwide to date, mainly in Japan (14, 15).

## Molecular and pathophysiological mechanisms

Plasma FXIII circulates as a heterotetramer composed of two A subunits and two B subunits (A_2_B_2_) held together by strong non-covalent interactions. The A subunits are encoded by the *F13A1* gene on chromosome 6p24.3–p25.3 and are synthesized in megakaryocytes and monocytes/macrophages, with hepatocytes reported as a minor additional source, whereas the B subunits are encoded by *F13B* gene on chromosome 1q31–q32.1 and are produced and secreted by hepatocytes. Structurally, the A subunits are 731-amino acid polypeptides with an acetylated N-terminal serine residue, within which the catalytic transglutaminase activity of the molecule resides. In its native state, the catalytic cysteine (Cys314) is inaccessible because it is shielded by a 37-amino acid N-terminal activation peptide (AP). The B subunits serve as carriers that stabilize the A subunits and are present in excess in the circulation (4, 16).

FXIII activation is initiated by thrombin-catalyzed cleavage at the Arg37–Gly38 site on the A subunit, releasing the AP. In the presence of calcium, the B subunits subsequently dissociate, reducing their affinity for the A subunits. This is followed by conformational changes in the cleaved FXIII-A_2_ that expose the catalytic region and active-site cysteine residues of the A subunits, rendering them functionally active and thereby forming the active transglutaminase FXIIIa. The presence of polymerized fibrin accelerates thrombin-catalyzed FXIII cleavage by up to 100-fold.

In addition to the circulating form, FXIII is present in the cytoplasm of hematopoietic cells, including platelets and monocytes, where it exists predominantly as A_2_ homodimers. For activation, elevating cellular calcium is sufficient to induce the conformational change. This intracellular pool contributes to thrombus formation following platelet activation and has been implicated in platelet-dependent processes, including adhesion (17).

FXIIIa is a transglutaminase that catalyzes acyl transfer from the γ-carboxamide group of glutamine residues (donors) to the ε-amino group of lysine residues (acceptors), resulting in the formation of ε-(γ-glutamyl)lysine covalent crosslinks with concurrent release of ammonia. Through this mechanism, FXIII exerts its principal physiological function of fibrin stabilization by catalyzing the formation of γ-chain dimers and high-molecular-weight α-chain polymers, thereby substantially enhancing the mechanical strength and structural integrity of the clot (4).

FXIIIa also incorporates α*2*-antiplasmin, the main physiological inhibitor of plasmin, into the fibrin network through a reaction involving Gln14 of α*2*-antiplasmin and Lys303 on the fibrin α chain, which enhances resistance to premature fibrinolysis. Beyond fibrin stabilization, FXIIIa facilitates interactions between fibrin(ogen) and extracellular matrix proteins such as fibronectin, vitronectin, and collagen, contributing to clot integration with surrounding tissue and supporting processes involved in tissue repair, including angiogenesis and re-epithelialization, and wound healing. This function is particularly relevant in pregnancy, where FXIII is essential for cytotrophoblast invasion and placental implantation (6, 7). Additionally, FXIII facilitates the incorporation of blood cells into the forming blood clot, an important step in clot retraction (18, 19).

Given the multifaceted role of FXIII and its interactions with several components, deficiency leads to a wide range of manifestations; deficiency of the cellular FXIII pool affects clot retraction and overall thrombus stability; defective fibrin crosslinking results in clots that are structurally weaker and more susceptible to breakdown, which manifests as soft-tissue bleeding; defective incorporation of α*2*-antiplasmin further contributes to premature fibrinolysis; and the loss of FXIII interactions with the extracellular matrix components may lead to delayed tissue repair, inadequate granulation tissue formation, an increased tendency for wound dehiscence, and early pregnancy loss (18).

Beyond deficiency states, elevated FXIII levels have been associated with arterial and thrombotic disorders; however, a detailed discussion of these associations is beyond the scope of this review (20, 21).

## Classification and clinical presentation

As more acquired forms of FXIIID are recognized, encompassing immune- and non-immune-mediated etiologies, the Subcommittee on Factor XIII and Fibrinogen of the International Society on Thrombosis and Haemostasis (ISTH) Scientific and Standardization Committee (SSC) is currently working on a project to further refine the classification of FXIII deficiencies (9, 22).

o **Congenital factor XIII deficiency**

A rare inherited bleeding disorder transmitted in an autosomal recessive mode. Most reported mutations are missense, with frameshift, splice-site, and nonsense mutations being less frequent. The majority of cases (∼95%) are due to mutations in the *F13A1* gene (OMIM #613225), leading to deficiency of the FXIII-A subunit and typically resulting in a more severe phenotype due to loss of the catalytic function.

*F13B* mutations (OMIM #613235) are far less common and typically result in a milder phenotype than F13A deficiency, largely because residual FXIII-A catalytic activity is often preserved. These mutations impair the carrier function of the B subunits, which prevents spontaneous activation of the A subunits. Hence, defects in the B subunits lead to instability and reduced levels of the FXIIIA_2_B_2_ complex (23).

Several factors influence disease severity, most importantly, allelic zygosity; individuals with severe disease carry homozygous or compound heterozygous mutations, as well as the type of mutation and its location within the protein, including the active site and subunit-interaction interfaces (24).

In general, severe congenital FXIIID is classically associated with FXIII activity levels below 5%, below which the risk of spontaneous major bleeding – including intracranial hemorrhage – becomes clinically significant. Data from the European Network of Rare Bleeding Disorders (EN-RBD) further suggest that FXIII activity levels ≥ 30% are sufficient for patients to remain asymptomatic, whereas levels <15% are associated with a substantially increased risk of grade III bleeding (21). The EN-RBD framework additionally proposes a three-tier severity classification based on registry-derived correlations between activity levels and bleeding phenotype: severe (undetectable activity, <1%), associated with spontaneous major bleeding; moderate (<30%), associated with a heterogeneous bleeding pattern ranging from post-traumatic or spontaneous minor mucocutaneous bleeding to spontaneous major hemorrhage; and mild (≥30%), which is most often asymptomatic (25, 26).

The mutational spectrum continues to expand as new variants are identified. To date, approximately 199 and 20 pathogenic variants have been reported in *F13A1* and *F13B*, respectively, as documented in the Human Gene Mutation Database (HGMD^1^) and the ISTH mutations registry^2^.

Congenital FXIII-A deficiency is further classified into two types based on the biochemical defect pattern:

∘ Type I FXIII-A deficiency: Quantitative disorder characterized by reductions in FXIII antigen and activity levels.
∘ Type II FXIII-A deficiency: Qualitative disorder characterized by normal to near normal levels of FXIII-A antigen levels with reduced activity levels.

Presentation: Heterozygous (carriers): Affected individuals are generally asymptomatic, with FXIII activity levels typically ranging from 20% to 60%. Bleeding upon hemostatic challenge – such as tooth extraction, intervention, or surgery – and obstetric complications may occasionally occur and can be significant. Some reports suggest a weaker or less predictable correlation between subnormal FXIII levels and bleeding manifestations in mild deficiency (10, 24).

Homozygous/compound heterozygous: In this severe form of the disease, there is a strong clinical correlation between residual FXIII activity and bleeding severity, with more severe manifestations associated with lower residual activity levels; severe deficiency is generally defined as FXIII activity below 5% (10, 25, 27). In the neonatal period, delayed umbilical stump bleeding – occurring days to weeks after delivery – is a classic clinical hallmark of severe FXIIID and may represent the earliest manifestation of the disorder, occurring in up to 80% of affected neonates (8). In affected males, post-circumcision bleeding may similarly be an early presenting feature. Intracranial hemorrhage, which can be spontaneous or traumatic, is the most serious complication, occurring in approximately 30% of untreated individuals predominantly during childhood (28), with risk substantially elevated when FXIII activity falls below 10%–15%. Other manifestations include bruising (60%), subcutaneous and intramuscular hematomas (55%), mucosal hemorrhage (30%), impaired wound healing with dehiscence and abnormal scar formation (14%–29%), delayed bleeding following hemostatic challenges – typically 12–36 h after the provoking event – excessive postoperative bleeding, and menorrhagia in affected females (9%) (8, 29–31). Unlike hemophilia, joint bleeding is relatively uncommon.

Pregnancy-related manifestations: The risk of adverse obstetric outcomes is significantly increased, including recurrent miscarriage, placental abruption, preterm delivery, antepartum hemorrhage, and postpartum hemorrhage (8, 13).

o **Acquired factor XIII deficiency**

A rare acquired bleeding diathesis, though it is more common than the congenital form, and can be caused by immune and non-immune causes.

Immune-mediated cases result from the development of autoantibodies that can neutralize or accelerate the clearance (non-neutralizing) of FXIII-A subunits or, less commonly, FXIII-B subunits (autoimmune FXIIID) (15). It usually coexists with other autoimmune conditions, such as systemic lupus erythematosus (∼30% of autoimmune cases), IgA vasculitis (Henoch–Schönlein purpura), and rheumatoid arthritis; it has also been associated with malignancies, certain medications–including penicillin, isoniazid, and phenytoin–and pregnancy (32, 33). However, it is idiopathic in up to 50% of cases, with a predilection for elderly patients. Immune-mediated cases typically manifest as “type II” FXIIID, characterized by reduced activity with normal or near-normal antigen levels.

Non-immune causes are more common than their immune-mediated counterparts (34), though likely underrepresented in registries and published case series, given their generally milder clinical phenotype and lower likelihood of formal diagnosis. They typically result in milder FXIII reductions (20%–70%) and a less severe bleeding diathesis, though clinically significant deficiency can still occur. Etiologies include consumptive states such as acute thrombosis, sepsis, inflammation, COVID-19, ulcerative colitis, and disseminated intravascular coagulation (DIC); dilutional coagulopathy; and decreased hepatic synthesis, which can occur in critically ill patients and with hepatic dysfunction (9).

Presentation: Autoimmune cases typically present with an acute onset of severe, often life-threatening bleeding, usually in the absence of prior bleeding manifestations or a known bleeding disorder. In contrast, non-immune acquired cases tend to result in milder FXIII reductions and a less severe bleeding diathesis; in most instances, the clinical picture is dominated by the manifestations of the underlying condition, such as sepsis, acute thrombosis, or DIC, rather than bleeding attributable to FXIIID *per se* (35).

## Diagnosis

A complete diagnosis of FXIIID requires a systematic approach encompassing a detailed personal and family bleeding history, with particular attention to bleeding episodes typical of the disorder, a thorough clinical evaluation, and a series of specialized laboratory investigations.

Currently, the ISTH SSC Subcommittee on Factor XIII and Fibrinogen has undertaken a project to identify appropriate first-line and confirmatory assays for laboratory FXIII testing and to define testing strategies for immune- and non-immune-mediated etiologies (9, 22).

FXIIID should be suspected in individuals with a suggestive bleeding history who have a normal coagulation screening profile, including prothrombin time (PT), activated partial thromboplastin time (aPTT), thrombin time (TT), platelet count, and fibrinogen, after more common entities – namely von Willebrand disease and platelet function disorders – have been excluded.

**FIGURE 1:**
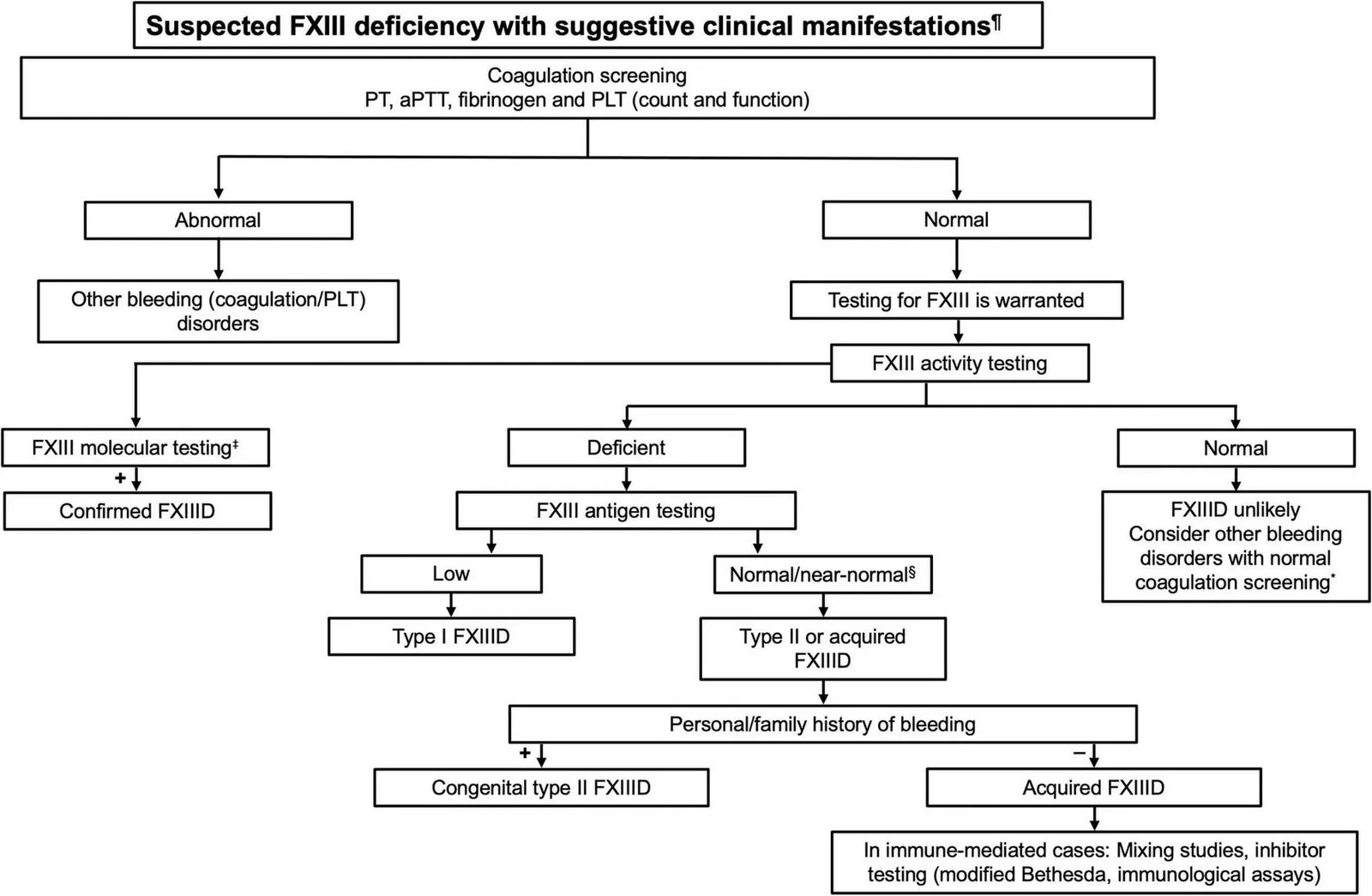
Laboratory approach for suspected factor XIII deficiency. aPTT, activated partial thromboplastin time; FXIII, factor XIII; FXIIID, factor XIII deficiency; PT, prothrombin time; PLT, platelets. ¶Classical (personal/family) bleeding manifestations include umbilical cord stump bleeding, intracranial hemorrhage, and recurrent miscarriage. ^‡^Although not required for diagnosis, molecular genetic testing may assist in family and reproductive counseling and in the identification of rare F13B-deficient cases, for whom recombinant FXIII-A_2_ is not an appropriate therapeutic option. *Bleeding disorders with a normal coagulation screen: Von Willebrand disease (unless factor VIII is sufficiently reduced to prolong the aPTT), acquired von Willebrand syndrome, hereditary hemorrhagic telangiectasia, vascular Ehlers-Danlos syndrome, and hyperfibrinolysis disorders (Alpha-2-antiplasmin deficiency and plasminogen activator inhibitor-1 deficiency). §With discordance between activity and antigen levels.

The laboratory approach to FXIIID is outlined in Figure 1 and encompasses the following strategies:

### Qualitative (functional) assays

The clot solubility test remains a widely used screening assay for FXIIID in many settings, owing to its simplicity, low cost, and lack of requirement for specialized reagents or technical expertise – including in approximately 20% of developed countries and more broadly across resource-limited settings in low- and middle-income countries (14). The test involves inducing clot formation by adding calcium, with or without thrombin, to plasma, then suspending the clot in a solubilizing agent (2% acetic acid, 1% monochloroacetic acid, or 5 mol/L urea). If FXIII activity is sufficient, the clot remains intact for up to 24 h; in FXIIID, it dissolves within minutes to hours (4). Historically, various modifications were attempted to improve assay performance, but did not yield clinically meaningful improvements in standardization and, in fact, made the assay more cumbersome and laborious. Consequently, they were not adopted in clinical practice (13). The clot solubility test carries several important limitations, including low specificity – with false-positive results in dys- and hypofibrinogenemia – poor sensitivity for mild-to-moderate deficiency, lack of standardization, and inability to quantify FXIII activity or antigen levels (36). Reliance on this assay risks underdiagnosis and precludes its use for monitoring replacement therapy. Accordingly, it is no longer recommended as a screening tool for FXIIID, except in resource-limited settings where validated quantitative assays are unavailable (11).

Fibrin crosslinking can also be assessed by gel electrophoresis (SDS-PAGE), which evaluates γ-chain dimerization and α-chain polymerization as qualitative or semi-quantitative measures of FXIIIa activity. However, given its cumbersome and time-consuming nature, it is considered an adjunctive research tool and is not recommended as part of the routine diagnostic workup (36).

### Quantitative (functional) assays

Three functional assays are currently available for FXIII activity testing: ammonia-release assays, amine-incorporation assays, and isopeptidase assays. These assays detect both quantitative and qualitative deficiencies and are recommended as first-line testing modality whenever available, a recommendation reflected in their widespread adoption (37). However, there are challenges and limitations to each of these assays, including low sensitivity and variation in the lower limit of quantitation (<1%–10%) (14).

1- Ammonia-release assays are among the most commonly used methods, including Berichrom^®^ FXIII (Siemens Healthcare Diagnostics) and Technochrom^®^ FXIII (Technoclone). The principle is based on measuring FXIII activity through a coupled enzymatic reaction. Following activation of FXIII by thrombin and calcium, the formed FXIIIa catalyzes crosslinking of a glutamine-containing oligopeptide substrate to glycine ethyl ester, releasing ammonia as a byproduct. The released ammonia is consumed in a glutamate dehydrogenase-coupled reaction, leading to the oxidation of a chromogen that absorbs light, NADH in the case of Berichrom^®^ and NADPH in the case of Technochrom^®^. The rate of absorbance decrease, measured spectrophotometrically at 340 nm, is proportional to FXIIIa activity (36). These assays are quick, easily automated, reproducible, and not influenced by the FXIII-A Val34Leu polymorphism (discussed further under ‘Diagnostic pitfalls’, subsection ‘Analytical’). However, a recognized limitation is that other plasma ammonia-generating reactions, independent of FXIII, can consume the chromogen, leading to an overestimation of FXIII activity, especially at FXIII activity levels < 20%. To overcome this, an iodoacetamide-containing blank should be included to inhibit FXIIIa and isolate the background signal; subtracting this from the test result yields the true FXIII-specific activity. This step is needed for the Berichrom^®^ assay, while the Technochrom^®^ reagent kit incorporates this blank directly (38). In practice, however, the blank correction step is not universally implemented, and many laboratories continue to perform the assay unmodified, in part due to safety concerns associated with iodoacetamide, which is classified as a chemical hazard (34). Furthermore, fibrinogen levels can influence FXIII activity measured by ammonia-release assays, where low levels can yield erroneously low factor activity (39), and the lower limit of quantification of approximately 3%–5% may limit reliable detection in severe deficiency states (9).

2- The amine incorporation assay measures FXIII activity by quantifying the covalent cross-linking of small-molecular-weight amine substrates – fluorescent, radiolabeled, or biotinylated – into glutamine residues of a protein substrate, via FXIIIa-catalyzed transamidation. Following FXIII activation, fibrin polymerization is inhibited by the addition of inhibitory peptides and further dilution of the test plasma. The reaction is subsequently stopped, and unbound amines are separated from the protein-bound fraction, which is then quantitatively measured as a reflection of FXIIIa activity. Although sensitive, this assay is time-consuming, difficult to standardize, sensitive to FXIII-A Val34Leu polymorphism (leading to erroneous overestimation), and unsuitable for true kinetic measurement due to the required separation step, limiting its routine clinical use. A biotinylated commercial version is available from Covalab; however, radiolabeled and fluorescent variants remain predominantly research tools (13).

3- The isopeptidase-based fluorogenic assay, Technofluor^®^ FXIII Activity (Technoclone), run on the Ceveron s100 analyzer (Technoclone), represents an alternative approach to measuring FXIII activity. First described by Parameswaran et al. in the late 1990s and subsequently modified (40, 41), it exploits the ability of FXIIIa to cleave isopeptide bonds contained within a fluorogenic substrate, the reverse of its physiological crosslinking function. The emitted fluorescence is proportional to FXIIIa activity in the test sample. As this method is independent of ammonia release, a plasma blank is not required. Additionally, the assay is rapid, direct, automatable, and specific, as the fluorogenic substrate is exclusively cleaved by thrombin-activated FXIIIa (39). However, despite these advantages, the assay has limited sensitivity, with a lower limit of quantification of ∼5% (14).

Generally, FXIII activity testing is performed on platelet-poor plasma with a platelet count below 10,000/μL to ensure accurate results (42). However, measurement of platelet lysate FXIII activity plays an important role in the subclassification of FXIIID. In inherited FXIII-A deficiency, platelet FXIII activity is reduced and remains low even during replacement therapy, whereas it is preserved in FXIII-B deficiency and in acquired FXIIID due to anti-FXIII autoantibodies. Platelet lysate, prepared by lysis with Triton X-100, is compatible with both ammonia-release and amine incorporation assay platforms (43).

### Antigen assays

Antigen assays are typically reserved as second-line investigations when activity levels are reduced, enabling accurate classification of the deficiency subtype. These assays are generally less widely available than functional assays and are typically performed in specialized hemostasis laboratories (26).

Antigen assays measure FXIII antigen levels independent of functional activity. In most instances, antigen and activity levels are concordant; however, clinically significant discordance (i.e., markedly reduced activity with relatively preserved antigen levels) may be observed in congenital type II deficiency and in acquired FXIIID due to neutralizing autoantibodies. A similar discordance pattern might be encountered in afibrinogenemia, representing an additional context in which concurrent measurement of both parameters is warranted (36, 44).

Factor XIII antigen levels are measured using immunological methods employing monoclonal or polyclonal antibodies directed against either the intact FXIII A_2_B_2_ heterotetramer or the individual subunits. Four main immunological assays have been described: enzyme-linked immunosorbent assays (ELISAs), radioimmunoassays (RIA), electroimmunoassays (EIA), and latex-enhanced immunoprecipitation assays, with varying sensitivities and specificities that largely depend on the antibodies used.

ELISA-based platforms are the most widely used for FXIII antigen quantification. Several types are in use, including the R-ELISA (Reanal-Ker), a one-step sandwich ELISA with sensitivity as low as 0.1% of normal, that is suitable for monitoring substitution therapy, and highly sensitive chemiluminescence FXIII antigen ELISA–with limits of detection of 0.014 μg/L for FXIII-A, 0.019 μg/L for FXIII-B, and 0.016 μg/L for FXIII-A_2_B_2_– (14, 45).

RIA and EIA are used less frequently due to lower sensitivity and greater technical complexity.

Automated latex-enhanced immunoassays are also commercially available as practical alternatives, including the HemosIL^®^ factor XIII antigen latex agglutination immunoassay (Instrumentation Laboratory) and the K-Assay^®^ FXIII (Stago). These assays quantify FXIII antigen (A-subunit for the HemosIL) using latex particles coated with anti-human polyclonal rabbit antibodies. Upon binding to FXIII in the test plasma, immune complexes form and cause light scatter, measured turbidimetrically at 500–600 nm; the resulting signal is proportional to the FXIII antigen concentration in the sample (14).

Despite the availability of assays targeting various FXIII components, some laboratories rely on platforms that quantify FXIII-A exclusively, potentially failing to detect rarer FXIII-B subunit deficiencies (14).

In efforts to harmonize FXIII measurement, the WHO 1^st^ International Standard for Factor XIII Plasma (NIBSC code 02/206) was established, with assigned values for plasma FXIII activity, FXIII-A_2_B_2_ antigen, and – following a subsequent collaborative study – FXIII-B subunit antigen, all expressed in International Units (IU/mL), providing a common reference framework for interlaboratory standardization (46, 47).

### Factor XIII inhibitor testing

Inhibitor assays are performed to differentiate congenital from acquired FXIIID caused by anti-FXIII autoantibodies and to detect anti-FXIII alloantibodies, which may develop in patients receiving FXIII replacement therapy and typically manifest as suboptimal post-infusion activity recovery or accelerated clearance.

Anti-FXIII antibodies are classified into two types: neutralizing and non-neutralizing. Neutralizing antibodies are detected by mixing studies, in which equal volumes of patient plasma and normal pooled plasma are combined, and FXIII activity is measured immediately and after incubation at 37 °C for 1–2 h. Although the Bethesda assay is not standardized for FXIII inhibitors, laboratories may adapt it as a laboratory-developed test to quantify the inhibitor once a neutralizing antibody is suspected. Non-neutralizing antibodies are detected by immunological methods such as ELISA, though such assays are not widely available (14, 48).

### Genetic testing

Although molecular testing is not mandatory for establishing the diagnosis of FXIIID, identification of the causative genetic variant can assist in family studies, carrier detection, and reproductive counseling, including prenatal genetic testing when appropriate. Additionally, identification of the causative mutation has important therapeutic implications, as recombinant FXIII-A_2_ (catridecacog) replaces only the A subunit and is therefore appropriate for cases with *F13A1* mutations but not for the less common *F13B* mutations, particularly when antigen testing is not routinely performed in the diagnostic laboratory (49).

High-throughput next-generation sequencing (NGS) panels encompassing *F13A1* and *F13B* can identify the underlying variants in most cases of congenital FXIIID. However, standard sequencing approaches may not detect all pathogenic alterations, mainly deep intronic variants, large structural rearrangements, or copy-number variants unless dedicated deletion/duplication analysis is included. Sanger sequencing remains useful for confirming identified variants and performing targeted testing for known familial variants (36).

### Global coagulation assays

Viscoelastic tests (VET), including thromboelastography and rotational thromboelastometry, assess global coagulation but have important diagnostic limitations in FXIIID. Their tracings predominantly reflect thrombin-mediated fibrin formation and overall clot kinetics; since FXIII primarily functions in the later phases of clot stabilization–enhancing clot firmness and resistance to fibrinolysis–reduced FXIII activity may not be captured. Consequently, VET parameters may yield normal tracings despite low FXIII activity levels.

Nevertheless, results may be abnormal or of clinical utility in cases of severe deficiency or during monitoring of FXIII replacement therapy, respectively. Experimental modifications incorporating exogenous fibrinolytic agents, such as tissue plasminogen activator, to evaluate clot susceptibility to lysis have been described (50, 51); however, these approaches are not standardized and are not currently recommended for the diagnostic evaluation of FXIIID (9, 11).

A comparative summary of FXIII laboratory testing modalities used in clinical practice is presented in Table 1.

**TABLE 1:** Comparison of laboratory assays for the diagnosis of factor XIII deficiency used in clinical practice.

Assay (category)	Assay/platform	Principle	Availability	Detects	LLOQ	Key advantages	Key limitations/pitfalls	Recommended role
Qualitative (functional)	Clot solubility test	Clot dissolution in urea, acetic acid, or monochloroacetic acid after thrombin/calcium-induced clot formation	Routine/resource-limited	Type I & II (severe only)	N/A	Simple; low cost; no specialized equipment; widely available	Low sensitivity and specificity; non-quantitative; not standardized; cannot monitor therapy	Resource-limited settings only; not recommended where quantitative assays are available
Quantitative (functional) first-line	Ammonia-release assays (Berichrom^®^ & Technochrom^®^)	Ammonia released during the FXIIIa transglutaminase reaction is consumed in a GDH-coupled reaction. The resultant decrease in NADH/NADPH absorbance is measured spectrophotometrically at 340 nm.	Routine/automated	Type I & II	∼3%–5%	Rapid; automatable; reproducible; unaffected by Val34Leu polymorphism	Background NH3 from other plasma reactions overestimates activity at <20%; (Berichrom) iodoacetamide blank correction required but not universally performed; result is affected by fibrinogen levels, LLOQ may limit reliable detection in severe deficiency	First-line; widely used
	Amine incorporation assay	Quantification of covalent incorporation of tagged amines into glutamine residues of a protein substrate, catalyzed by FXIIIa	Specialized/research		Variable	Sensitive; detects all FXIIID forms	Time-consuming; difficult to standardize; sensitive to Val34Leu polymorphism (overestimates); requires separation step; not suitable for kinetic measurement	Research; limited routine use
	Isopeptidase fluorogenic assay Technofluor^®^ FXIII activity (Technoclone/Ceveron s100)	Quantification of emitted fluorescence upon cleavage of isopeptide bonds within a fluorogenic substrate by FXIIIa	Specialized/automated		∼5%	No plasma blank required; rapid; direct; automatable; specific for thrombin-activated FXIIIa; unaffected by NH3 background	Limited sensitivity; LLOQ ∼5%; single platform availability	First-line (where available)
Antigen testing second-line	ELISA	Immunological quantification using antibodies directed against FXIII-A, FXIII-B, or A_2_B_2_ heterotetramer	Specialized	Type I In type II: normal/near normal	R-ELISA: 0.1% (chemiluminescence ELISA):FXIII-A: 0.014 μg/L; FXIII-B: 0.019 μg/L; A_2_B_2_: 0.016 μg/L	High sensitivity; subunit-specific quantification; can detect FXIII-B deficiency when B-subunit assay used	Not widely available; some platforms measure FXIII-A only, missing FXIII-B deficiency; requires specialized laboratory	Second-line (after reduced activity confirmed); subtype classification
	Latex agglutination immunoassay HemosIL^®^ FXIII antigen (Instrumentation Laboratory); K-Assay^®^ FXIII (Stago)	Latex particles coated with anti-FXIII antibodies; immune complex formation causes light scatter measured turbidimetrically at 500–600 nm	Routine/automated		2.5% and 2.3%, respectively		(HemosIL): measures FXIII-A only – will not detect isolated FXIII-B deficiency	
Inhibitor testing	Mixing study	Mixing of equal volumes of patient and NPP; FXIII activity measured immediately and after 37 °C incubation for 1–2 h	Specialized	Neutralizing anti-FXIII antibodies	N/A	Distinguishes congenital from acquired immune-mediated deficiency; detects alloantibodies post-replacement	Screening test only; does not quantify inhibitor strength; result interpretation requires expertise	When acquired immune-mediated deficiency suspected; post-replacement inhibitor workup
	(Bethesda-adapted, laboratory-developed test)	Serial dilutions of patient plasma mixed with NPP; residual FXIII activity vs. control converted to titer via Bethesda-style calibration (not FXIII-validated)	Specialized		N/A		Not standardized for FXIII; laboratory-developed test; result interpretation requires expertise	
	Anti-FXIII ELISA	Immunological detection of anti-FXIII antibodies	Specialized	Non-neutralizing anti-FXIII antibodies	N/A	Detects antibodies not identified by mixing studies	Not widely available	
Genetic testing	NGS; Sanger sequencing	Sequencing of *F13A1* and *F13B* coding regions	Specialized/molecular lab	Detection of causative variants confirms diagnosis	N/A	Identifies causative variant in most congenital cases; may influence therapeutic choice	Not required for diagnosis; not available in all centers; (NGS) may miss deep intronic variants, large structural rearrangements, or CNVs without dedicated analysis	Genetic/reproductive counseling; carrier detection; prenatal testing; subtype confirmation when F13B deficiency suspected

CNVs, copy number variants; ELISA, enzyme-linked immunosorbent assay; FXIII, coagulation factor XIII; FXIIIa, activated factor XIII; FXIIID, factor XIII deficiency; GDH, glutamate dehydrogenase; LLOQ, lower limit of quantitation; NADH/NADPH, nicotinamide adenine dinucleotide (phosphate); NGS, next-generation sequencing; NH3, ammonia; NPP, normal pool plasma.

## Diagnostic pitfalls

Unlike other coagulation factors that are part of the intrinsic, extrinsic, or common pathways, whose deficiencies produce characteristic abnormalities detectable by routine coagulation screening, FXIII exerts its catalytic activity at the terminal step of the coagulation cascade, after fibrin polymerization has already occurred. Accordingly, standard coagulation screening is typically normal in FXIIID. Diagnosis, therefore, requires dedicated FXIII assays; however, these are not widely available, and even when accessible, they may go unrequested given the rarity of the condition.

Beyond test availability and ordering practices, FXIII diagnosis faces further challenges at both the preanalytical and analytical levels.

### Preanalytical

As FXIII-A is present in platelet alpha granules, platelet-poor plasma – ideally obtained by double centrifugation – is required for testing, to avoid potential overestimation of plasma FXIII activity.

### Analytical

There is considerable inter-individual variability in baseline FXIII activity, partly attributable to common coding polymorphisms in the *F13A1* gene, which complicates the definition of a universal reference range and may contribute to misclassification of mild deficiency states (14). The most clinically relevant of these is the Val34Leu polymorphism, with a substantially variable prevalence across ethnic groups – ranging from almost absent in some Asian populations to approximately 44% in Caucasians – which accelerates FXIII activation up to 2.5-fold without altering the specific activity of fully activated FXIIIa. Consequently, amine incorporation assays that measure transglutaminase activity during the early activation phase may overestimate FXIII activity in carriers of the Leu34 allele, whereas ammonia-release assays, including Berichrom^®^ and Technochrom^®^, measure fully activated FXIIIa and are unaffected by this polymorphism (13).

### Clinical implications of Val34Leu polymorphism

Beyond the aforementioned analytical implications, the Leu34 allele has been associated with a modest protective effect against venous thromboembolism and, to a lesser extent, coronary artery disease (52, 53). Despite these reported protective associations, current evidence is insufficient to support routine Val34Leu genotyping in thrombophilia evaluation, and no major international thrombophilia guideline recommends its inclusion in standard thrombophilia testing panels (54, 55). Nevertheless, the interaction between Val34Leu and other thrombophilic risk factors, as well as the potential implications of genotype for the interpretation of selected FXIII assays, warrant continued investigation.

## Management

The primary goal of management in congenital FXIIID is the prevention of spontaneous hemorrhage, particularly intracranial bleeding, and the provision of hemostatic cover during trauma, surgical interventions, and childbirth. Given the relatively long plasma half-life of FXIII (approximately 9–14 days), extended dosing intervals of up to 4 weeks are widely used in clinical practice. Therapy should, however, be individualized based on FXIII activity levels and clinical response.

### Prophylaxis therapy

Prophylaxis significantly reduces the rate of spontaneous bleeding and intracranial events in severe congenital FXIIID (4, 48). Given the risk of serious and potentially fatal hemorrhagic complications, which may occur unpredictably, prophylactic FXIII supplementation initiated early in life is strongly recommended and has become the standard of care in persons with severe deficiency (30).

Plasma-derived FXIII (pdFXIII) concentrate, Fibrogammin^®^ P: Europe/Corifact^®^: USA (CSL Behring) remains the most commonly used first-line replacement therapy for prophylaxis and peri-interventional management of congenital FXIIID. The product is well tolerated and effective in preventing and treating bleeding in individuals with congenital FXIIID, with a plasma half-life of ∼7–9 days (26, 56). An initial intravenous dose of 40 IU/kg is administered with subsequent dosing adjusted to maintain trough FXIII activity within the range of 5%–20%, repeated at approximately 28-days intervals (57). Data from the Prospective Rare Bleeding Disorders Database (PRO-RBDD) study propose a FXIII activity level of 15% as the minimum threshold for initiating prophylaxis, below which the risk of spontaneous major bleeding increases (from 50% to >90% for FXIII levels of 15% and ≤5%, respectively) (27).

Recombinant human FXIII-A_2_, catridecacog: NovoThirteen^®^: Europe/Tretten^®^: USA (Novo Nordisk), is a protransglutaminase consisting of two FXIII-A subunits (rA_2_ homodimer) that is produced through transgene expression in *Saccharomyces cerevisiae*. When delivered to the circulation, it binds to free human FXIII B-subunit, forming a heterotetramer (rA_2_B_2_) with a half-life of ∼11 days (58). It is approved by the U.S. Food and Drug Administration and the European Medicines Agency and several other regulatory authorities for routine prophylaxis of bleeding in patients with congenital FXIII A-subunit deficiency (*F13A1* mutations), though it confers no benefit in the setting of FXIII-B subunit deficiency (*F13B* mutations) (58, 59). It has a favorable efficacy and safety profile established across the mentor™ clinical trial program (26); In the pivotal mentor™1 trial, a phase 3 open-label study, monthly intravenous prophylaxis at 35 IU/kg resulted in a markedly reduced annualized bleeding rate (ABR) of 0.138, compared with 2.91 historically observed under on-demand treatment. All treatment-requiring bleeding episodes were trauma-induced, and no spontaneous bleeds or intracranial hemorrhages were recorded throughout the trial period. These findings were further corroborated and extended in the mentor™2 long-term extension trial, the largest clinical dataset in congenital FXIIID to date, encompassing 186.5 patient-years of exposure, in which the mean ABR declined further to 0.043, with a mean spontaneous ABR of only 0.011 (60–62).

### On-demand therapy

On-demand therapy is indicated in the management of acute bleeding episodes and for hemostatic coverage during surgical or invasive procedures. Both pdFXIII and rFXIII-A_2_ may be used, though rFXIII-A_2_ is only appropriate in patients with F13A1 deficiency. PdFXIII is administered intravenously at an initial dose of 20–40 IU/kg, with subsequent dosing guided by post-infusion FXIII activity monitoring. Target FXIII activity levels of 20%–30% are generally recommended for minor to moderate procedures, with higher targets applied for major surgical interventions (35).

### FXIII replacement-related adverse effect

Both plasma-derived and recombinant FXIII replacement products carry a favorable tolerability profile, with adverse effects reported at a low incidence. The most commonly reported adverse reactions in clinical trials (≥1%) include headache, leucopenia, joint inflammation, skin reactions (rash, pruritus, and erythema), increased lactate dehydrogenase, and pain in the extremities and at the injection site (58). More serious but infrequent adverse events include hypersensitivity reactions, thromboembolic events, and the development of neutralizing inhibitory antibodies. Of these, inhibitor development – though extremely rare –represents the most clinically significant complication, as it may render replacement therapy ineffective and precipitate life-threatening bleeding (43). Inhibitor development should be suspected in any patient demonstrating suboptimal FXIII recovery following infusion or a demonstrable shortening of the plasma half-life, and warrants prompt investigation to guide therapeutic decisions (63).

Plasma-derived FXIII is prepared from human plasma and thus undergoes rigorous pathogen mitigation steps throughout the manufacturing process, including nucleic acid screening, fractionation partitioning, and pasteurization. These measures are effective against enveloped viruses, such as HIV and hepatitis B and C viruses, as well as non-enveloped viruses, including hepatitis A virus and parvovirus B19. Nonetheless, the possibility of infection transmission cannot be entirely excluded, particularly with respect to emerging pathogens and agents not currently addressed by existing manufacturing steps.

As a precautionary measure, hepatitis A and B vaccination should be considered for patients who regularly receive plasma-derived FXIII products (64).

Given that rFXIII is manufactured without the use of human- or mammalian-derived materials, it carries no risk of pathogen transmission (viruses or prions) (65). This advantage has led to its adoption as the preferred treatment option in several countries, particularly across Europe (66).

It should be noted that no long-term animal studies evaluating the carcinogenicity, genotoxicity, or effects on fertility of rFXIII have been conducted; however, a dedicated carcinogenic risk assessment suggests minimal risk associated with its clinical use (58).

### Adjunctive and alternative hemostatic strategies

Antifibrinolytic therapy with tranexamic acid may be sufficient for minor bleeds, particularly those involving mucosal surfaces, and may serve as a useful adjunct to FXIII concentrate in more significant bleeding episodes. It should be noted, however, that tranexamic acid is relatively contraindicated in renal tract bleeding and in patients with elevated thrombotic risk. In the perioperative setting, prophylactic administration – either orally or intravenously – is recommended approximately 2 h prior to surgery (26).

Alternative therapies, when FXIII concentrate is unavailable, include cryoprecipitate and fresh-frozen plasma (FFP), though both carry important limitations. The FXIII content in these products is highly variable across units and batches, making it difficult to predict the expected activity increment. Moreover, achieving sufficient FXIII levels often requires larger infusion volumes, raising the risk of transfusion-associated circulatory overload (TACO), especially with FFP, and allergic reactions. Where these alternatives must be used, a pragmatic approach – broadly analogous to general factor replacement strategies – would involve FFP at 10–20 mL/kg or cryoprecipitate at approximately 1 unit per 5–10 kg body weight, with post-infusion FXIII activity measurement to guide further dosing. In resource-limited settings, periodic small-volume plasma infusions have been employed, taking advantage of the relatively long half-life of FXIII.

Platelet concentrates have also been identified as a potential emergency source of FXIII when no other product is available. However, platelets contain only intracellular FXIII-A, and their contribution to plasma FXIII activity is minimal and unpredictable; they should be regarded as a measure of last resort (26, 67).

Available FXIII replacements are summarized in Table 2.

**TABLE 2:** Recombinant and plasma-derived products available for factor XIII replacement.

Product	Product composition	Indication	Strength/vial content	Contraindications	Special scenarios
Corifact, Fibrogammin pdFXIII	Lyophilized powder of purified human pd FXIII concentrate containing both A- and B-subunits water for reconstitution (4 or 20 ml) (Fibrogammin) contains 124.4–195.4 mg (5.41–8.50 mmol) sodium per dose (Corifact) contains 140–220 mg sodium chloride per vial.^§^	Congenital FXIII deficiency (A and B subunits); prophylaxis and perioperative management	(Small vial): 250 IU (Large vial): 1250 IU	Known hypersensitivy/anaphylactic reactions to human plasma-derived products Cautious use in case of acute thrombosis^¶^	In case of hypersensitivity during infusion, e.g., hives, urticaria, tightness of the chest, and hypotension, infusion should be discontinued immediately. In case of shock, current medical standards for shock protocol should be immediately implemented.^——^
Catridecacog (NovoThirteen) rFXIII-A	2500 IU powder, and 3 ml solvent	FXIII A-subunit deficiency	2500 IU	Known hypersensitivity to the product	Do not administer with recombinant factor VIIa due to risk of thrombosis Pregnancy: NovoThirteen may be considered during pregnancy only if clearly indicated; no adequate human data available Not effective in FXIII B-subunit deficiency For pediatric dosing: for patients weighing < 24 kg, reconstituted NovoThirteen should be diluted with 6 mL of sodium chloride 0.9%
Catridecacog (Tretten) rFXIII-A	White lyophilized powder and a diluent; sterile water (3.2 ml)		2500 IU per single-use vial (2000–3125 IU)* After reconstitution, each vial contains 667–1042 IU/mL rFXIII	Known hypersensitivity to the product	Do not administer with rfVIIa; risk of thrombosis Pregnancy: No human or animal data available. Use only if clearly needed.
Cryoprecipitate	Cold-insoluble precipitate derived from FFP; contains fibrinogen (>150 mg/unit), FVIII (>80 IU/unit), VWF, FXIII (40–60 IU/unit), and fibronectin	Alternative when FXIII concentrates unavailable	FXIII content ∼2.5–3 IU/mL^‡^	Known hypersensitivity to plasma products; relative contraindication in patients at risk of TACO	Due to the variable FXIII content, post-infusion FXIII activity measurement is recommended (85)
FFP	Whole plasma containing all coagulation factors at normal plasma concentrations, including FXIII		FXIII content ∼1 IU/mL^‡^	Known hypersensitivity to plasma products; IgA deficiency with anti-IgA antibodies; high risk of TACO	Larger volumes required to achieve therapeutic FXIII levels; requires ABO compatibility (86)

ABO, ABO blood group system; FFP, fresh-frozen plasma; FVIII, factor VIII; FXIII, factor XIII; IV, intravenous; pdFXIII, plasma-derived FXIII; rFXIII-A, recombinant coagulation factor XIII; TACO, transfusion-associated circulatory overload; VWF, von Willebrand factor. *The actual amount of Tretten in IU is stated on each carton and vial. ^§^ To be considered in patients on a controlled sodium diet. ^¶^ Caution is advised in cases of acute thrombosis, given the fibrin-stabilizing effect of FXIII. ^‡^Content is variable between units and batches; in clinical practice, cryoprecipitate is commonly dispensed as a pool of 4–6 units rather than as a single unit. ^——^Shorter half-life and faster clearance in patients < 16 years; dose adjustments may be needed. All listed products are administered intravenously.

### Special scenarios

#### Neonatal/pediatric management

Compared with adults, pediatric patients, especially at very young ages, often exhibit faster plasma clearance and reduced half-life of administered coagulation factors such as recombinant FXIII (68), which may necessitate more frequent dosing to maintain adequate trough activity levels; weight-based dosing considerations further complicate management in this population. However, the mentor™4 trial, which assessed the pharmacokinetics (PK) and safety of recombinant FXIII in children aged 1 to ≤6 years with congenital FXIII A-subunit deficiency, demonstrated that a standard dose of 35 IU/kg, similar to the established dose in adults (60, 61), maintained plasma FXIII activity above the critical threshold of 10% throughout the 28-days dosing interval, confirming that no age-based dose adjustment is required across pediatric and adult populations (48). These findings were further corroborated by the mentor™5 trial, a phase 3b open-label study in which the same six children received continued monthly rFXIII-A_2_ prophylaxis for a minimum of 52 weeks. Throughout the study period, no treatment-requiring bleeding episodes occurred, yielding an ABR of zero, and the geometric mean FXIII trough activity was consistently maintained at 19%. No antibody development, thromboembolic events, or allergic reactions were observed, collectively reinforcing the safety and efficacy of rFXIII-A_2_ prophylaxis in this particularly vulnerable age group (30).

In the perioperative setting, higher activity thresholds are generally recommended to ensure adequate hemostasis (4).

#### Gynecologic and obstetric management

Heavy menstrual bleeding can be managed by hormonal therapies (e.g., combined oral contraceptives or levonorgestrel-releasing intrauterine devices), which are highly effective first-line interventions. Adjunct therapies, such as antifibrinolytics (e.g., tranexamic acid), are frequently required to achieve hemostatic control (69).

Antenatal care for women with FXIIID requires close multidisciplinary collaboration, involving obstetrics, hematology, transfusion medicine, and anesthesia, with ongoing communication with the hematology laboratory to ensure timely monitoring throughout pregnancy. Management should be individualized based on disease severity and clinical response (4).

Due to the rarity of the disease and the lack of robust clinical evidence, current pregnancy and peripartum management is largely derived from observational data and expert opinion.

As FXIII is essential for both hemostasis and successful maintenance of pregnancy (e.g., placental development and pregnancy preservation), replacement therapy is indicated throughout pregnancy in women with severe congenital FXIIID.

Women with severe FXIII-A deficiency cannot carry a pregnancy to term without FXIII replacement therapy (43). Available evidence suggests a pragmatic approach of fortnightly prophylaxis to maintain FXIII trough levels of ≥10%–20% in early pregnancy, with higher trough levels (i.e., ≥30%) in late pregnancy and early peripartum period. During vaginal deliveries and cesarean sections, the suggested dosing is 10%–40% IU/kg of plasma-derived FXIII to prevent bleeding (70).

Regarding anesthesia administration, a careful risk-benefit analysis should always be undertaken, given the potential for neuraxial hematoma. Available evidence suggests that neuraxial anesthesia can be performed safely when FXIII activity is maintained at ≥30%, provided there is no additional coagulopathy (71, 72).

Prenatal diagnosis is recommended, particularly in consanguineous parents. This can be done with invasive tests such as chorionic villus sampling or amniocentesis performed at 11–14 and after 15 weeks’ gestation, respectively, and non-invasive prenatal tests (NIPT) done as early as 9–10 weeks’ gestation. The risks and benefits of each approach, particularly for invasive procedures, should be carefully discussed with the family prior to testing.

Affected fetuses are at increased risk of bleeding during delivery and labor, especially from invasive monitoring and assisted vaginal deliveries. Intracranial hemorrhage is the most deleterious complication, which can potentially lead to long-term neurological deficit or death. Planned C-section has the lowest risk of bleeding complications in the newborn and thus can be offered for affected or potentially affected fetuses with severe FXIIID (73). Accordingly, delivery should occur at a tertiary center with clinical and laboratory expertise in FXIIID and access to hemostatic therapy. These newborns should undergo prompt evaluation for congenital FXIIID, with consideration of early initiation of prophylaxis if severe deficiency is confirmed (69).

Post-partum hemorrhage (PPH) represents an additional serious complication with significant morbidity and mortality risks. Antifibrinolytic therapy is a key preventive and therapeutic component in PPH. Tranexamic acid, administered either intravenously or orally, at the time of delivery and then continued postpartum, significantly reduces the risk of PPH and maternal mortality (69).

#### Management of acquired FXIII deficiency

Management of immune-mediated acquired FXIIID can be difficult and is largely influenced by the titer and kinetics of anti-FXIII autoantibodies. Although FXIII replacement has been attempted, the response has been inconsistent. In practice, management is built around two main pillars (74). The first is controlling bleeding and maintaining hemostasis, usually with general supportive measures and antifibrinolytic agents. The second pillar is eradicating the inhibitor, which remains the cornerstone of treatment in acquired cases.

Initial therapy generally involves corticosteroids, either alone or combined with rituximab, with responses often observed within 4–8 weeks. Individuals who do not respond may benefit from other immunosuppressive agents, such as cyclophosphamide or mycophenolate mofetil. The duration of therapy is ideally guided by serial measurements of FXIII activity and inhibitor levels, starting with weekly monitoring and gradually extending the intervals as improvement is seen (75).

It is also important to identify and manage any underlying triggers, including autoimmune diseases, malignancies, or culprit medications. Ongoing follow-up is recommended, as relapse remains a recognized risk (75, 76).

In non-immune-mediated acquired FXIIID, patient management should be individualized and guided by bleeding severity, concurrent coagulopathies, required interventions, and comorbidities. Treatment of the precipitating cause should be the primary approach, and factor replacement is not routinely required. In the perioperative setting, however, where acquired FXIIID is a recognized and clinically relevant complication, FXIII administration has been shown to ameliorate bleeding by correcting hyperconsumption-driven reductions in FXIII activity (77).

**FIGURE 2:**
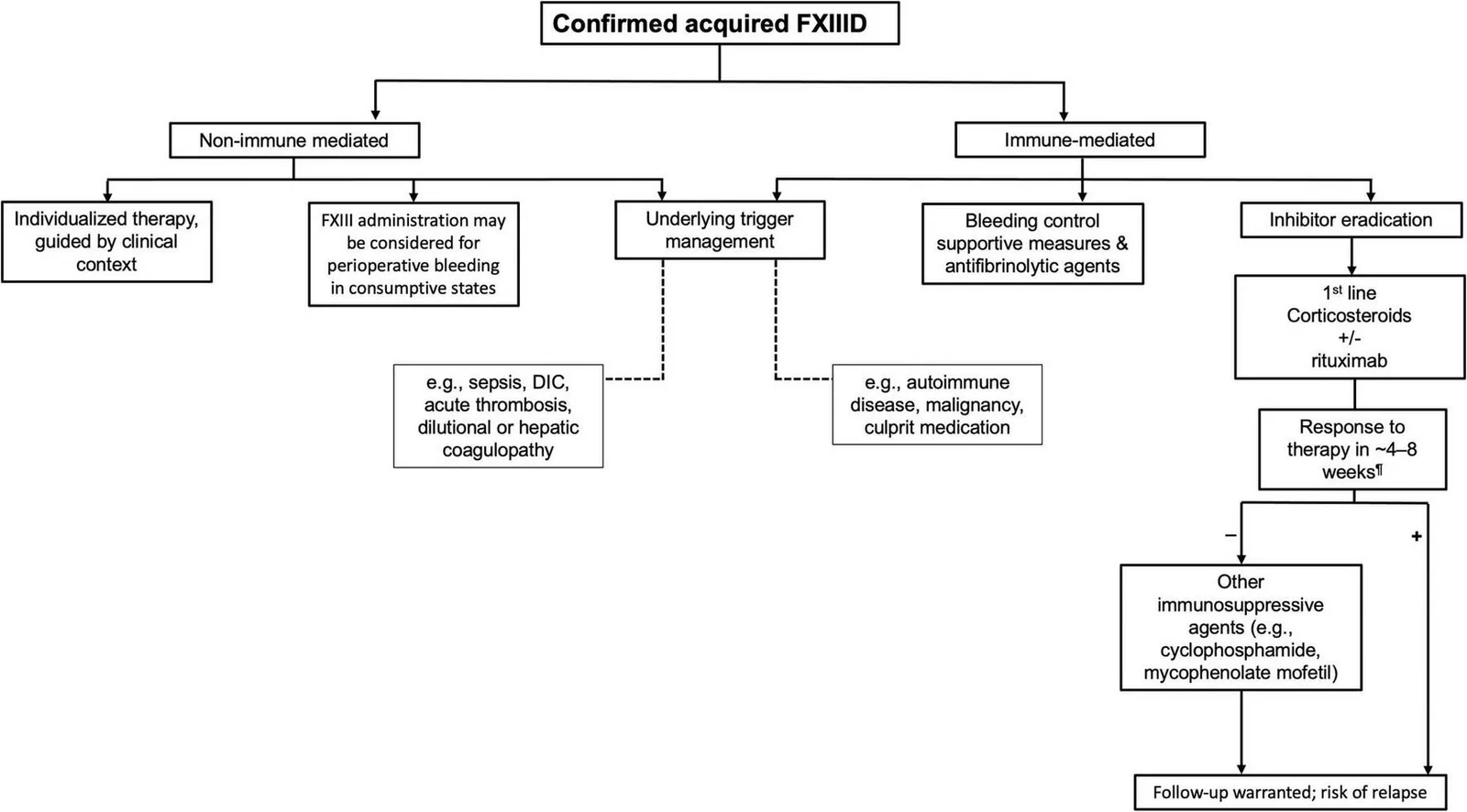
Therapeutic approach to acquired factor XIII deficiency. DIC, disseminated intravascular coagulation; FXIII, factor XIII; FXIIID, factor XIII deficiency. ¶Response to therapy is guided by serial measurements of FXIII activity and inhibitor levels.

The therapeutic approach to acquired FXIII deficiency is summarized in Figure 2.

#### Anticoagulation in the setting of FXIII deficiency

While thrombotic events in congenital FXIIID are exceedingly rare, their management presents a unique clinical challenge, as anticoagulation must be carefully balanced against the underlying hemorrhagic diathesis. No specific guidelines exist for anticoagulation in this setting. Insights from the management of more prevalent congenital bleeding disorders, particularly hemophilia, may inform clinical decision-making, albeit with important caveats. In patients with hemophilia, current recommendations are to maintain a factor trough level of 20% for oral anticoagulation, with direct oral anticoagulation preferred over vitamin K antagonists, and emphasize that anticoagulation should not be initiated in patients with severe hemophilia without concurrent clotting factor prophylaxis (78, 79). However, it is uncertain if this threshold can be applied to persons with FXIIID, given FXIII’s distinct roles (hemostatic and non-hemostatic) compared to factors VIII and IX. The only published experience on the use of anticoagulation in FXIIID comes from Bounaix et al., who described the first reported case of severe congenital FXIIID complicated by recurrent venous thromboembolic events, managed with rivaroxaban alongside plasma-derived FXIII replacement without bleeding complications. Their approach involved a stepwise reduction in replacement intensity based on the anticoagulation phase: trough FXIII activity was targeted at 50% during the rivaroxaban loading phase, reduced to 30% during maintenance, and further lowered to 20% during long-term prophylactic dosing. The authors concluded that rivaroxaban appears safe in this setting, provided that the intensity of replacement therapy is appropriately adjusted (80). Given the very limited evidence, management decisions should be highly individualized, guided by close monitoring of FXIII activity levels, and ideally reached through shared decision-making within a multidisciplinary framework.

#### Emerging and future therapeutic strategies

Gene therapy represents an attractive long-term therapeutic strategy for congenital FXIIID, as replacement of a single defective gene could potentially provide sustained endogenous FXIII production; however, clinical development remains substantially less advanced than in hemophilia, and human data are currently lacking. Individualized pharmacokinetic-guided dosing, in which replacement regimens are tailored to patient-specific FXIII recovery, clearance, bleeding phenotype, and lifestyle factors, is another promising approach, with precision medicine strategies that may optimize factor utilization while maintaining adequate hemostatic protection. Finally, development of even longer-acting FXIII formulations may further reduce infusion frequency and improve treatment convenience. Nevertheless, given the established long half-life of currently available products and the effectiveness of monthly prophylactic regimens, such approaches remain largely conceptual at present. Beyond replacement-based strategies, non-factor rebalancing therapies that enhance thrombin generation, several of which have successfully entered clinical practice in hemophilia, are unlikely to correct the core defect in FXIIID, as the primary pathological lesion lies in fibrin cross-linking and clot stabilization rather than in impaired thrombin generation (81, 82).

## Prognosis

**Congenital factor XIII deficiency:** In untreated individuals, serious hemorrhagic complications occur in up to 66.7% of patients with FXIII activity below 10 IU/dL (83), with intracranial hemorrhage (ICH) being among the most serious complications and has been reported in approximately 25%–30% of untreated patients (43). Moreover, spontaneous central nervous system bleeding is the primary cause of death in affected patients (27). Additionally, in 50% of survivors, post-ICH neurological complications can occur, most commonly motor disability (58). Obstetric outcomes are similarly affected in untreated women, with all pregnancies in the absence of prophylaxis resulting in miscarriage, compared with a miscarriage rate of 18.8% in those receiving prophylactic replacement.

**Acquired factor XIII deficiency:** Nonimmune cases are associated with high rates of bleeding and prolonged intensive care unit stays. Additionally, among ICU patients, FXIII activity <50% is associated with a higher number of red cell transfusions (34, 35). Immune-mediated cases carry a substantially higher mortality, reported at approximately 25% within the first year of diagnosis (43, 84).

## Conclusion and future directions

Congenital and acquired FXIIID are rare but clinically significant disorders associated with potentially life-threatening bleeding complications, with intracranial hemorrhage being the most severe, as well as adverse obstetric outcomes and impaired wound healing. Diagnosis is challenging because routine coagulation screening assays cannot detect this deficiency. This diagnostic challenge is further compounded by limited assay availability, underrecognition of the condition in clinical practice, and a number of preanalytical and analytical pitfalls, including poor sensitivity of widely utilized assays in resource-limited areas with a potential of underdiagnosis of mild/moderate cases and the confounding influence of the Val34Leu polymorphism on potential overestimation of activity in select assays. The consequence, not uncommonly, is a delayed diagnosis at the cost of preventable morbidity and mortality. Addressing this requires not only appropriate laboratory investigation – anchored by quantitative FXIII activity assays and supplemented by antigen measurement, inhibitor testing, and genetic characterization where relevant – but also, and perhaps more fundamentally, a sustained clinical awareness of the condition.

In congenital deficiency, the advent of FXIII concentrates has genuinely changed the natural history of the disorder, with consistent prophylaxis effectively eliminating the spontaneous and intracranial bleeding that once defined it. Special management scenarios, including pregnancy, the peripartum period, pediatric patients, and the rare individuals who develop thrombotic complications requiring anticoagulation, remain challenging and largely consensus-driven rather than evidence-based; bridging these evidence gaps will require deliberate investment in prospective international registries and collaborative research. Acquired FXIIID, whether immune- or nonimmune- mediated, remains an underappreciated clinical entity requiring recognition and individualized management.

Future advances in gene therapy, pharmacokinetic-guided precision dosing, and extended-duration FXIII replacement products may further reduce treatment burden and improve long-term outcomes; yet the more immediate priority remains closing the gap between the availability of effective treatment and the recognition of the patients who need it.

## Footnotes

1
https://www.hgmd.cf.ac.uk

2
https://www.isth.org/page/MutationsRareBleedin

## References

[1] Robbins KC. A study on the conversion of fibrinogen to fibrin. *Am J Physiology-Legacy Content.* (1944) 142:581–8. 10.1152/ajplegacy.1944.142.4.581

[2] Lorand L Jacobsen A. Studies on the polymerization of fibrin; the role of the globulin: fibrin-stabilizing factor. *J Biol Chem.* (1958) 230:421–34. 10.1016/S0021-9258(18)70577-2 13502411

[3] Laki K Lóránd L. On the solubility of fibrin clots. *Science.* (1948) 108:280. 10.1126/science.108.2802.280 17842715

[4] Jacobs JW Booth GS Costa V Figueroa Villalba CA Savani BN Adkins BD. Factor XIII deficiency: a review of biology, testing, and treatment. *Clin Hematol Int.* (2026) 8:10–25. 10.46989/001c.155180 41583548 PMC12825037

[5] Schroeder V Durrer D Meili E Schubiger G Kohler HP. Congenital factor XIII deficiency in Switzerland: from the worldwide first case in 1960 to its molecular characterisation in 2005. *Swiss Med Wkly.* (2007) 137:272–8. 10.4414/smw.2007.11756 17594539

[6] Dorgalaleh A. Novel insights into heterozygous factor XIII deficiency. *Semin Thromb Hemost.* (2024) 50:200–12. 10.1055/s-0043-1764471 36940714

[7] Aljabry M. Factor XIII deficiency in the Saudi population, an underestimated bleeding risk. review article and an illustrative case report with dental complications. *Saudi Dent J.* (2023) 35:305–9. 10.1016/j.sdentj.2023.03.015 37251713 PMC10213848

[8] Karimi M Bereczky Z Cohan N Muszbek L. Factor XIII deficiency. *Semin Thromb Hemost.* (2009) 35:426–38. 10.1055/s-0029-1225765 19598071

[9] Kohler HP Ichinose A Seitz R Ariens RA Muszbek L. Diagnosis and classification of factor XIII deficiencies. *J Thromb Haemost.* (2011) 9:1404–6. 10.1111/j.1538-7836.2011.04315.x 22946956

[10] Singh S Pezeshkpoor B Jamil MA Dodt J Sharma A Ramar V et al. Heterozygosity in factor XIII genes and the manifestation of mild inherited factor XIII deficiency. *J Thromb Haemost.* (2024) 22:379–93. 10.1016/j.jtha.2023.09.032 37832789

[11] Mumford AD Ackroyd S Alikhan R Bowles L Chowdary P Grainger J et al. Guideline for the diagnosis and management of the rare coagulation disorders: a United Kingdom Haemophilia Centre Doctors’ Organization guideline on behalf of the British Committee for Standards in Haematology. *Br J Haematol.* (2014) 167:304–26. 10.1111/bjh.13058 25100430

[12] Dorgalaleh A Naderi M Hosseini MS Alizadeh S Hosseini S Tabibian S et al. Factor XIII deficiency in Iran: a comprehensive review of the literature. *Semin Thromb Hemost.* (2015) 41:323–9. 10.1055/s-0034-1395350 25615432

[13] Katona É Pénzes K Molnár É Muszbek L. Measurement of factor XIII activity in plasma. *Clin Chem Lab Med.* (2012) 50:1191–202. 10.1515/cclm-2011-0730 22850052

[14] Karimi M Peyvandi F Naderi M Shapiro A. Factor XIII deficiency diagnosis: challenges and tools. *Int J Lab Hematol.* (2018) 40:3–11. 10.1111/ijlh.12756 29027765

[15] Ichinose A. Autoimmune acquired factor XIII deficiency due to anti-factor XIII/13 antibodies: a summary of 93 patients. *Blood Rev.* (2017) 31:37–45. 10.1016/j.blre.2016.08.002 27542511

[16] Komáromi I Bagoly Z Muszbek L. Factor XIII: novel structural and functional aspects. *J Thromb Haemost.* (2011) 9:9–20. 10.1111/j.1538-7836.2010.04070.x 20880254

[17] Mitchell JL Mutch NJ. Novel aspects of platelet factor XIII function. *Thromb Res.* (2016) 141:S17–21. 10.1016/S0049-3848(16)30356-5 27207415

[18] Opneja A Kapoor S Stavrou EX. Contribution of platelets, the coagulation and fibrinolytic systems to cutaneous wound healing. *Thromb Res.* (2019) 179:56–63. 10.1016/j.thromres.2019.05.001 31078121 PMC6556139

[19] Muszbek L Bereczky Z Bagoly Z Komáromi I Katona É. Factor XIII: a coagulation factor with multiple plasmatic and cellular functions. *Physiol Rev.* (2011) 91:931–72. 10.1152/physrev.00016.2010 21742792

[20] Bagoly Z Koncz Z Hársfalvi J Muszbek L. Factor XIII, clot structure, thrombosis. *Thromb Res.* (2012) 129:382–7. 10.1016/j.thromres.2011.11.040 22197181

[21] Muszbek L Bereczky Z Bagoly Z Shemirani AH Katona E. Factor XIII and atherothrombotic diseases. *Semin Thromb Hemost.* (2010) 36:18–33. 10.1055/s-0030-1248721 20391293

[22] International Society on Thrombosis and Haemostasis *Updated Recommendations for the Diagnosis and Classification of Factor XIII Deficiencies.* (2025). Available online at: https://my.isth.org/communities/community-home?CommunityKey=5958ee15-1418-4d67-8159-8df8fad7ff5c (accessed 14 April, 2025).

[23] Ivaskevicius V Seitz R Kohler HP Schroeder V Muszbek L Ariens RA et al. International registry on factor XIII deficiency: a basis formed mostly on European data. *Thromb Haemost.* (2007) 97:914–21. 10.1160/TH07-01-0034 17549292

[24] Naderi M Dorgalaleh A Alizadeh S Tabibian S Hosseini S Sadat Hosseini M et al. Clinical manifestations and bleeding episodes among heterozygote individuals of factor XIII deficiency, a short term prospective study. *Blood.* (2014) 124:2852. 10.1182/blood.V124.21.2852.2852

[25] Peyvandi F Palla R Menegatti M Siboni SM Halimeh S Faeser B et al. Coagulation factor activity and clinical bleeding severity in rare bleeding disorders: results from the European Network of Rare Bleeding Disorders. *J Thromb Haemost.* (2012) 10:615–21. 10.1111/j.1538-7836.2012.04653.x 22321862

[26] Peyvandi F Di Michele D Bolton-Maggs PH Lee CA Tripodi A Srivastava A. Classification of rare bleeding disorders (RBDs) based on the association between coagulant factor activity and clinical bleeding severity. *J Thromb Haemost.* (2012) 10:1938–43. 10.1111/j.1538-7836.2012.04844.x 22943259

[27] Menegatti M Palla R Boscarino M Bucciarelli P Muszbek L Katona E et al. Minimal factor XIII activity level to prevent major spontaneous bleeds. *J Thromb Haemost.* (2017) 15:1728–36. 10.1111/jth.13772 28688221

[28] Alavi SER Jalalvand M Assadollahi V Tabibian S Dorgalaleh A. Intracranial hemorrhage: a devastating outcome of congenital bleeding disorders-prevalence, diagnosis, and management, with a special focus on congenital factor XIII deficiency. *Semin Thromb Hemost.* (2018) 44:267–75. 10.1055/s-0037-1604109 28898896

[29] Ichinose A. Factor XIII is a key molecule at the intersection of coagulation and fibrinolysis as well as inflammation and infection control. *Int J Hematol.* (2012) 95:362–70. 10.1007/s12185-012-1064-3 22477542

[30] Kerlin BA Inbal A Will A Williams M Garly ML Jacobsen L et al. Recombinant factor XIII prophylaxis is safe and effective in young children with congenital factor XIII-A deficiency: international phase 3b trial results. *J Thromb Haemost.* (2017) 15:1601–6. 10.1111/jth.13748 28581691

[31] Al-Sharif FZ Aljurf MD Al-Momen AM Ajlan AM Musa MO Al-Nounou RM et al. Clinical and laboratory features of congenital factor XIII deficiency. *Saudi Med J.* (2002) 23:552–4. 10.15537/1658-3175.1704 12070580

[32] Haroon A Mohammed Saleh MF Alahmari A Osman S Alotaibi A Alzahrani H et al. Acquired factor XIII deficiency in myeloid neoplasms: case series and review of literature. *Hematol Oncol Stem Cell Ther.* (2024) 17:245–7. 10.4103/hemoncstem.hemoncstem-D-24-00034 39829100

[33] Duranteau O Tatar G Demulder A Tuna T. Acquired factor XIII deficiency: a scoping review. *Eur J Anaesthesiol Intensive Care.* (2023) 2:e0035. 10.1097/EA9.0000000000000035 39916809 PMC11783664

[34] Al Sharif MA Mathews N Tasneem S Moffat KA Carlino SA Mithoowani S et al. Measurement of factor XIII for the diagnosis and management of deficiencies: insights from a retrospective review of 10 years of data on consecutive samples and patients. *Res Pract Thromb Haemost.* (2025) 9:102689. 10.1016/j.rpth.2025.102689 40027442 PMC11871450

[35] Duque P Chasco-Ganuza M Ortuzar A Almaraz C Terradillos E Perez-Rus G et al. Acquired FXIII deficiency is associated with high morbidity. *Thromb Haemost.* (2022) 122:48–56. 10.1055/a-1481-2733 33851388

[36] Schroeder V. Laboratory assessment of coagulation factor XIII. *Hamostaseologie.* (2020) 40:467–71. 10.1055/a-1181-0327 32869231

[37] Jennings I Kitchen S Menegatti M Palla R Walker I Makris M et al. Detection of factor XIII deficiency: data from multicentre exercises amongst UK NEQAS and PRO-RBDD project laboratories. *Int J Lab Hematol.* (2017) 39:350–8. 10.1111/ijlh.12633 28406553

[38] Cini M Legnani C Frascaro M Pancani C Cappelli C Rodorigo G et al. Measurement of factor XIII (FXIII) activity by an automatic ammonia release assay using iodoacetamide blank-procedure: no more overestimation in the low activity range and better detection of severe FXIII deficiencies. *Clin Chem Lab Med.* (2016) 54:805–9. 10.1515/cclm-2015-0547 26457781

[39] Leitner M Büchold C Pasternack R Binder NB Moore GW. Clinical validation of an automated fluorogenic factor XIII activity assay based on isopeptidase activity. *Int J Mol Sci.* (2021) 22:1002. 10.3390/ijms22031002 33498248 PMC7863959

[40] Oertel K Hunfeld A Specker E Reiff C Seitz R Pasternack R et al. highly sensitive fluorometric assay for determination of human coagulation factor XIII in plasma. *Anal Biochem.* (2007) 367:152–8. 10.1016/j.ab.2007.05.011 17582378

[41] Parameswaran KN Cheng XF Chen EC Velasco PT Wilson JH Lorand L. Hydrolysis of gamma:epsilon isopeptides by cytosolic transglutaminases and by coagulation factor XIIIa. *J Biol Chem.* (1997) 272:10311–7. 10.1074/jbc.272.15.10311 9092583

[42] CLSI *Collection, Transport, and Processing of Blood Specimens for Testing Plasma-Based Coagulation Assays and Molecular Hemostasis Assays; Approved Guideline.* Wayne, PA: CLSI (2008). Report No.: H21-A5.

[43] Muszbek L Katona É. Diagnosis and management of congenital and acquired FXIII deficiencies. *Semin Thromb Hemost.* (2016) 42:429–39. 10.1055/s-0036-1572326 27071048

[44] Bridey F Négrier C Duval C Ariëns R de Moerloose P Casini A. Impaired factor XIII activation in patients with congenital afibrinogenemia. *Haematologica.* (2019) 104:e111–3. 10.3324/haematol.2018.203901 30262556 PMC6395312

[45] Orosz ZZ Katona E Facskó A Berta A Muszbek LA. highly sensitive chemiluminescence immunoassay for the measurement of coagulation factor XIII subunits and their complex in tears. *J Immunol Methods.* (2010) 353:87–92. 10.1016/j.jim.2010.01.001 20079358

[46] Raut S Merton RE Rigsby P Muszbek L Seitz R Ariëns RA et al. A collaborative study to establish the 1st International Standard for factor XIII plasma. *J Thromb Haemost.* (2007) 5:1923–9. 10.1111/j.1538-7836.2007.02684.x 17723131

[47] Raut S Katona É Riches-Duit A Coxon C Muszbek L Schroeder V et al. An international collaborative study to assign value for Total Factor XIII-B subunit antigen to the WHO 1st International Standard for Factor XIII Plasma, (02/206): communication from the ISTH SSC Subcommittee on Factor XIII and Fibrinogen. *J Thromb Haemost.* (2022) 20:525–31. 10.1111/jth.15596 34784091

[48] Williams M Will A Stenmo C Rosholm A Tehranchi R. Pharmacokinetics of recombinant factor XIII in young children with congenital FXIII deficiency and comparison with older patients. *Haemophilia.* (2014) 20:99–105. 10.1111/hae.12224 23834599

[49] Javed H Singh S Ramaraje Urs SU Oldenburg J Biswas A. Genetic landscape in coagulation factor XIII associated defects - Advances in coagulation and beyond. *Blood Rev.* (2023) 59:101032. 10.1016/j.blre.2022.101032 36372609

[50] Rossetto A Torres T Platton S Vulliamy P Curry N Davenport RA. new global fibrinolysis capacity assay for the sensitive detection of hyperfibrinolysis and hypofibrinogenemia in trauma patients. *J Thromb Haemost.* (2023) 21:2759–70. 10.1016/j.jtha.2023.05.005 37207863

[51] Kuiper GJ Kleinegris MC van Oerle R Spronk HM Lancé MD Ten Cate H et al. Validation of a modified thromboelastometry approach to detect changes in fibrinolytic activity. *Thromb J.* (2016) 14:1. 10.1186/s12959-016-0076-2 26770073 PMC4712545

[52] Tang LV Morange PE Corral J Ntaios G Spyropoulos AC Lip GYH et al. Practical guideline for major hereditary thrombophilia. *Innovation.* (2025) 6:100890. 10.1016/j.xinn.2025.100890 40528894 PMC12169261

[53] Wells PS Anderson JL Scarvelis DK Doucette SP Gagnon F. Factor XIII Val34Leu variant is protective against venous thromboembolism: a HuGE review and meta-analysis. *Am J Epidemiol.* (2006) 164:101–9. 10.1093/aje/kwj179 16740590

[54] Middeldorp S Nieuwlaat R Baumann Kreuziger L Coppens M Houghton D James AH et al. American Society of Hematology 2023 guidelines for management of venous thromboembolism: thrombophilia testing. *Blood Adv.* (2023) 7:7101–38. 10.1182/bloodadvances.2023010177 37195076 PMC10709681

[55] Arachchillage DJ Mackillop L Chandratheva A Motawani J MacCallum P Laffan M. Thrombophilia testing: a British Society for haematology guideline. *Br J Haematol.* (2022) 198:443–58. 10.1111/bjh.18239 35645034 PMC9542828

[56] Brackmann HH Egbring R Ferster A Fondu P Girardel JM Kreuz W et al. Pharmacokinetics and tolerability of factor XIII concentrates prepared from human placenta or plasma: a crossover randomised study. *Thromb Haemost.* (1995) 74:622–5. 10.1055/s-0038-1649787 8584996

[57] Nugent D. Corifact™/Fibrogammin§P in the prophylactic treatment of hereditary factor XIII deficiency: results of a prospective, multicenter, open-label study. *Thromb Res.* (2012) 130:S12–4. 10.1016/S0049-3848(13)70005-7 23439001

[58] U.S. Food and Drug Administration *TRETTEN (coagulation factor XIII A-subunit, recombinant): Prescribing Information.* Silver Spring, MD: U.S. Food and Drug Administration (2013).

[59] European Medicines Agency *NovoThirteen (catridecacog): Summary of Product Characteristics.* Amsterdam: European Medicines Agency (2012).

[60] Carcao M Altisent C Castaman G Fukutake K Kerlin BA Kessler C et al. Recombinant FXIII (rFXIII-A_2_) prophylaxis prevents bleeding and allows for surgery in patients with congenital FXIII A-Subunit deficiency. *Thromb Haemost.* (2018) 118:451–60. 10.1055/s-0038-1624581 29448295 PMC6260112

[61] Inbal A Oldenburg J Carcao M Rosholm A Tehranchi R Nugent D. Recombinant factor XIII: a safe and novel treatment for congenital factor XIII deficiency. *Blood.* (2012) 119:5111–7. 10.1182/blood-2011-10-386045 22451421

[62] Poulsen LH Kerlin BA Castaman G Molinari AC Menegatti M Nugent D et al. Safety and effectiveness of recombinant factor XIII-A_2_ in congenital factor XIII deficiency: real-world evidence. *Res Pract Thromb Haemost.* (2022) 6:e12628. 10.1002/rth2.12628 35243202 PMC8882239

[63] Solomon C Korte W Fries D Pendrak I Joch C Gröner A et al. Safety of factor XIII concentrate: analysis of more than 20 years of pharmacovigilance data. *Transfus Med Hemother.* (2016) 43:365–73. 10.1159/000446813 27781024 PMC5073543

[64] CSL Behring GmbH, *Fibrogammin 250/1250 IU: Summary of Product Characteristics: Electronic Medicines Compendium (emc).* (2018). Available online at: https://www.medicines.org.uk/emc/product/3736/smpc (accessed May 2, 2026).

[65] Lovejoy AE Reynolds TC Visich JE Butine MD Young G Belvedere MA et al. Safety and pharmacokinetics of recombinant factor XIII-A_2_ administration in patients with congenital factor XIII deficiency. *Blood.* (2006) 108:57–62. 10.1182/blood-2005-02-0788 16556896

[66] Di Minno G Perno CF Tiede A Navarro D Canaro M Güertler L et al. Current concepts in the prevention of pathogen transmission via blood/plasma-derived products for bleeding disorders. *Blood Rev.* (2016) 30:35–48. 10.1016/j.blre.2015.07.004 26381318 PMC7115716

[67] O’Shaughnessy DF Atterbury C Bolton Maggs P Murphy M Thomas D Yates S et al. Guidelines for the use of fresh-frozen plasma, cryoprecipitate and cryosupernatant. *Br J Haematol.* (2004) 126:11–28. 10.1111/j.1365-2141.2004.04972.x 15198728

[68] Björkman S Oh M Spotts G Schroth P Fritsch S Ewenstein BM et al. Population pharmacokinetics of recombinant factor VIII: the relationships of pharmacokinetics to age and body weight. *Blood.* (2012) 119:612–8. 10.1182/blood-2011-07-360594 22042695

[69] Connell NT Davies J Abdul-Kadir R Kaplan Z. *Obstetric and Gynaecologic Considerations in Inherited Bleeding Disorders.* Montreal, QC: Haemophilia (2026). 10.1111/hae.70267 41988968

[70] Sharief LA Kadir RA. Congenital factor XIII deficiency in women: a systematic review of literature. *Haemophilia.* (2013) 19:e349–57. 10.1111/hae.12259 23992439

[71] Yi Y Niu B Duffett L El-Chaâr D Tinmouth A Wang TF et al. Neuraxial analgesia in pregnant individuals with bleeding disorders: a retrospective descriptive study of obstetric anesthesia practices and outcomes. *Res Pract Thromb Haemost.* (2025) 9:103186. 10.1016/j.rpth.2025.103186 41208942 PMC12590423

[72] Carroll DB Myler C Songdej N Sedeek K Bezinover D. Management of neuraxial analgesia in a parturient with factor XIII deficiency: a case report and proposed management algorithm. *Case Rep Anesthesiol.* (2020) 2020:8892225. 10.1155/2020/8892225 33489380 PMC7790575

[73] Davies J Kadir RA. Mode of delivery and cranial bleeding in newborns with haemophilia: a systematic review and meta-analysis of the literature. *Haemophilia.* (2016) 22:32–8. 10.1111/hae.12726 25990680

[74] Tone KJ James TE Fergusson DA Tinmouth A Tay J Avey MT et al. Acquired factor XIII inhibitor in hospitalized and perioperative patients: a systematic review of case reports and case series. *Transfus Med Rev.* (2016) 30:123–31. 10.1016/j.tmrv.2016.04.001 27167905

[75] Beckman JD Kasthuri RS Wolberg AS Ma AD. Challenges in diagnosis and management of acquired factor XIII (FXIII) inhibitors. *Haemophilia.* (2018) 24:e417–20. 10.1111/hae.13603 30144219 PMC6390178

[76] Song J Liu L Ding B Xia A Liu J Han Y et al. Comparison of outcomes in autoimmune acquired factor XIII deficiency with and without underlying diseases: a systematic review. *J Thromb Thrombolysis.* (2026) 59:116–24. 10.1007/s11239-025-03148-5 40745406

[77] Duque P Korte W. Factor XIII in the acute care setting and its relevance in obstetric bleeding. *Transfus Med Hemother.* (2022) 50:10–7. 10.1159/000526489 36818773 PMC9912001

[78] Escobar M Lassila R Bekdache C Owaidah T Sholzberg M. Use of antithrombotic therapy in patients with hemophilia: a selected synopsis of the European Hematology Association - International Society on Thrombosis and Haemostasis - European Association for Hemophilia and Allied Disorders - European Stroke Organization Clinical Practice Guidance document. *J Thromb Haemost.* (2025) 23:745–9. 10.1016/j.jtha.2024.10.033 39571935

[79] Schutgens REG Jimenez-Yuste V Escobar M Falanga A Gigante B Klamroth R et al. Antithrombotic treatment in patients with hemophilia: an EHA-ISTH-EAHAD-ESO clinical practice guidance. *Hemasphere.* (2023) 7:e900. 10.1097/HS9.0000000000000900 37304933 PMC10256340

[80] Bounaix L Schroeder V Fontana P Casini A. Management of anticoagulation and factor XIII replacement in a patient with severe factor XIII deficiency and recurrent venous thromboembolic disease: case report and review of literature. *Res Pract Thromb Haemost.* (2024) 8:102371. 10.1016/j.rpth.2024.102371 38595334 PMC11002293

[81] Palla R Peyvandi F Shapiro AD. Rare bleeding disorders: diagnosis and treatment. *Blood.* (2015) 125:2052–61. 10.1182/blood-2014-08-532820 25712993

[82] Peyvandi F Garagiola I Biguzzi E. Advances in the treatment of bleeding disorders. *J Thromb Haemost.* (2016) 14:2095–106. 10.1111/jth.13491 27590165

[83] Bouttefroy S Meunier S Milien V Boucekine M Chamouni P Desprez D et al. Congenital factor XIII deficiency: comprehensive overview of the FranceCoag cohort. *Br J Haematol.* (2020) 188:317–20. 10.1111/bjh.16133 31414482

[84] Franchini M Frattini F Crestani S Bonfanti C. Acquired FXIII inhibitors: a systematic review. *J Thromb Thrombolysis.* (2013) 36:109–14. 10.1007/s11239-012-0818-3 23065324

[85] Stanford S Roy A Cecil T Hegener O Schulz P Turaj A et al. Differences in coagulation-relevant parameters: comparing cryoprecipitate and a human fibrinogen concentrate. *PLoS One.* (2023) 18:e0290571. 10.1371/journal.pone.0290571 37647278 PMC10468048

[86] Green L Bolton-Maggs P Beattie C Cardigan R Kallis Y Stanworth SJ et al. British society of haematology guidelines on the spectrum of fresh frozen plasma and cryoprecipitate products: their handling and use in various patient groups in the absence of major bleeding. *Br J Haematol.* (2018) 181:54–67. 10.1111/bjh.15167 29527654

